# Engineering of Rotational Dynamics via Polymorph Manipulation

**DOI:** 10.1021/acs.jpca.4c04964

**Published:** 2024-12-05

**Authors:** Alfred Błażytko, Marzena Rams-Baron, Maria Książek, Joachim Kusz, Marek Matussek, Joanna Grelska, Marian Paluch

**Affiliations:** † August Chelkowski Institute of Physics, University of Silesia in Katowice, 75 Pulku Piechoty 1, 41-500 Chorzow, Poland; ‡ Institute of Chemistry, University of Silesia in Katowice, Szkolna 9, 40-006 Katowice, Poland

## Abstract

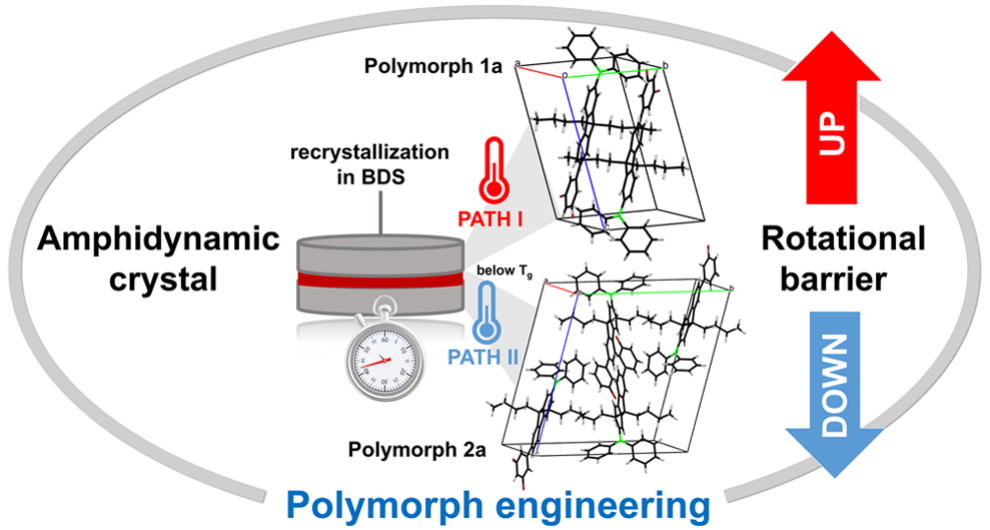

We used dielectric spectroscopy to uncover the rotational dynamics of the fluorophenyl rotor in different polymorphs of two amphidynamic crystals with identical sizable cores. The rotor solid-state dynamics were investigated in various crystalline environments. We did not change the chemical structure of the crystal itself, but while maintaining the same atomic composition, we changed the arrangement of atoms in space by taking advantage of crystal polymorphism, providing an alternative approach to one based on searching for new, chemically different entities with desirable functionality. We demonstrated that via polymorph variation, we can efficiently improve rotor solid-state performance and reduce the rotational barrier height by 30%. Our findings advance the understanding of polymorph engineering as a prospective trend in amphidynamic crystal technology, which uses the phenomenon of crystal polymorphism to design crystals displaying applicable internal rotational dynamics.

## Introduction

Molecular machines are increasingly attracting the attention of scientists and engineers, as their research and design contribute to the development of innovative materials with applications in various scientific and technological fields.^1−4^ One of the most rapidly developed types of molecular machines are molecular rotors, which are characterized by their ability to perform intramolecular rotational motions.^4−6^

In general, molecular rotation is a phenomenon involving the rotation of a molecule or a fragment of a molecule around its center of mass. Various molecular factors can affect the rotational behavior, including the molecular mass, shape of the molecule, or inter- and intramolecular interactions.^7−9^ In addition, the rotational image will vary notably depending on the state of matter. For instance, in the liquid phase, molecules have a lot of freedom to perform rotational motion with the time scale of relaxation times reaching nanoseconds.^10−12^ Liquid rotational dynamics are strongly temperature-dependent, so as the temperature decreases, a slowdown in molecular dynamics is observed. In some cases, it is possible to supercool the liquid and smoothly enter a disordered glassy state. In a glass, the whole molecule’s motion reaches a laboratory-inaccessible time scale. Still, the mobility of molecular fragments can be experimentally observed (if the structure of the molecule and its environment allows it).^13^ In other cases, liquid cooling can lead to crystal formation, where the mobility of molecules is precluded by a highly dense and well-ordered environment. Exceptions include plastic crystals, in which the centers of mass of the molecules are stationary, but the molecules can perform rotational movements.^7,12^ Another exception is the so-called amphidynamic crystals. In such structures, the static part of the molecule (the stator) remains trapped in the crystal structure, while the mobile fragment (the rotor) exhibits some rotational freedom to perform rotary motions.^14−16^

The key direction of research on amphidynamic crystals and the first step to create new paradigms in molecular rotor engineering is identifying relationships between the crystal structure and the rotational barrier height. Up to now, researchers’ efforts toward designing optimal rotational behavior have focused on examining newly synthesized compounds. An alternative unexplored area is to use polymorphic engineering for this purpose. It is unclear to what extent small changes in the arrangement of atoms in different polymorphs of one compound can affect the rotor performance. As such, crystal polymorphism in rotor engineering has not been fully utilized.

The leading and widely used technique in the study of rotation in crystals is nuclear magnetic resonance (NMR); this technique has some limitations, including a relatively small range of available relaxation times (about five decades).^17−21^ An alternative, increasingly used in polar rotor research, is broadband dielectric spectroscopy (BDS). It offers a much more comprehensive frequency range of up to 16 decades; thus, molecular mobility can be followed over a wide temperature range.^12,22^ For an intriguing class of amphidynamic crystals with polar rotors, the BDS method is an effective and convenient tool for studying their internal dynamics in a crystalline environment.

Motivated by the lack of general guidelines indicating the role of crystal polymorphism on the rotor’s performance in amphidynamic crystals, we used dielectric spectroscopy to investigate the internal rotational dynamics of two amphidynamic crystals with different polymorphs. We used two custom-designed systems containing a large, rigid stator and a fluorophenylene rotor rotating around a single carbon–carbon bond. We observed that depending on the applied crystallization protocol (involving different thermal steps), we could obtain distinct polymorphs of each compound. The combination of XRD and BDS methods allowed us to indicate how the polymorph variation impacts the rotational behavior of the fluorophenylene rotor in different crystal forms of two compounds. We believe that the results presented in this paper will advance the understanding of polymorph engineering as an emerging trend in amphidynamic crystal technology, which uses the phenomenon of crystal polymorphism to design crystals with useful intrinsic rotary dynamics.

**Figure 1:**
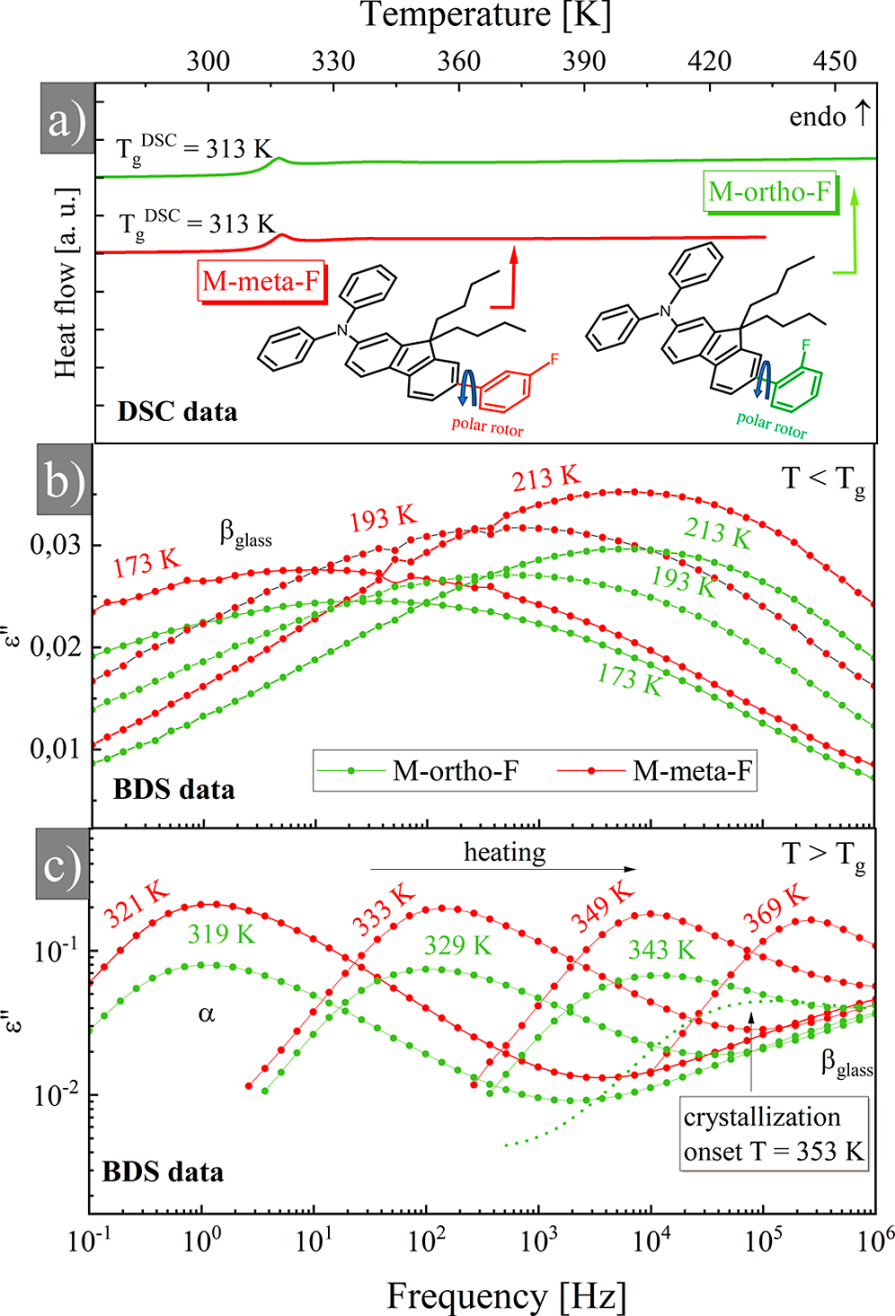
(a) Differential scanning calorimetry (DSC) thermograms measure on heating (10 K/min) of M-*meta*-F (red line) and M-*ortho*-F (green line). The chemical structures of both isomers are presented. Dielectric permittivity spectra ε″(*f*) collected for M-*meta*-F (red color) and M-*ortho*-F (green) in the glassy (b) and supercooled liquid (c) states. Symbols denote experimental data while solid lines are fitting functions. A spectrum plotted with a dashed line was partially crystalline.

Recently, we have proposed a new platform of crystalline molecular rotors with a sizable stator providing sufficient free volume for the solid-state rotation of a fluorophenylene unit.^23^ We demonstrated that via internal rotation, the rotor can sample the immediate surroundings with high sensitivity to molecular-level changes that impact the rotation parameters.^23,24^ These unique properties are determined by the presence of a fluorine atom, whose small size and electron-withdrawing nature allows for robust interaction with the rotor’s surroundings via C–H···F–C contacts. Previously investigated molecules have an acetylene linker between the stator and rotor, allowing fast rotary motion of the fluorophenyl moiety to be achieved in a crystalline environment with a barrier of 25.3 kJ/mol. To realize our concept of obtaining different polymorphs of sizable crystals, here, we replace the previously used acetylene linker with a single bond, which, although it limits the freedom of rotation, allows us to obtain different polymorphs of amphidynamic crystals, which was a central point of the current research. The chemical structure of two investigated molecules, referred to as M-*meta*-F (with 1,3-fluorophenylene rotor) and M-*ortho*-F (with 1,2-fluorophenylene rotor), is shown in Figure 1. These isomers differ only in the position of the fluorine atom in the rotating unit. The fluorophenylene rotor is connected by a single bond to a large, rigid, nonpolar part of a molecule containing diphenylamine and fluorene with long alkyl chains attached.

## Materials and Methods

### Materials

For our study, we used two structural isomers, i.e., M-*meta*-F (*N*-(7-(3-fluorophenyl)-9,9-tetrabutyl-9*H*-fluoren-2-yl)-*N*,*N*-diphenylamine) and M-*ortho*-F ((*N*-(7-(2-fluorophenyl)-9,9-tetrabutyl-9*H*-fluoren-2-yl)-*N*,*N*-diphenylamine). Both molecules were synthesized on request by TriMen Chemicals (Lodz, Poland) with a declared purity of >97%. The molecular mass of these compounds was *M* = 539.72 g/mol. Their structural formulas are presented in Figure 1a. Chemical characterization (^1^H NMR, ^13^C NMR, HRMS spectra) confirming the identity and purity of both compounds is presented in the Supporting Information (Figures S1–S6).

### Differential Scanning Calorimetry

Thermal analysis was performed by using a Mettler-Toledo calorimeter equipped with a ceramic HSS8 sensor (heat flux sensor with 120 thermocouples) and a liquid nitrogen cooling system. Temperature and enthalpy calibrations were performed by using indium and zinc standards. The sample was placed in 40 μL aluminum vessels. Samples in the amorphous and crystalline states were tested at 253–463 K for M-*ortho*-F and 253–433 K for M-*meta*-F with a heating rate of 10 K/min. The melting temperatures were determined from the onset of the process, while the glass transition temperature was assessed from the midpoint of the heat capacity change.

### Broadband Dielectric Spectroscopy

We performed dielectric measurements using a Novocontrol GMBH Alpha spectrometer. The Novocool Cryosystem system was responsible for temperature stabilization with an accuracy of 0.2 K. Most of the measurements were carried out in the frequency range from 10^–1^ to 3 × 10^6^ Hz. For temperature-dependent measurements of M-*ortho*-F polymorphs, the range was extended to 1 × 10^–2^ Hz. The sample in the amorphous state was placed between two stainless steel capacitor plates. A gap of 0.1 mm was provided by silica fibers. Dielectric loss spectra ε″(*f*) were measured on heating from 173 to 383 K for M-*meta*-F and from 173 to 359 K for M-*ortho*-F. The temperature step was 5 K for *T* < *T*_g_ and 2 K for *T* > *T*_g_. To obtain crystalline forms of the investigated isomers, we recrystallized samples from the supercooled liquid. Samples annealing at *T*_annealing_ > *T*_g_ was performed directly between the capacitor plates and the dielectric response was monitored as a function of time during this process. To obtain different polymorphic forms, we used two temperature protocols. The first involved heating the substance to *T*_m_ (*T* = 415 K and *T* = 440 K for M-*meta*-F and M-*ortho*-F, respectively), then cooling to *T*_annealing_ = 373 K and isothermal recrystallization at this temperature. In contrast, the second protocol involved heating to *T*_m_, cooling to 273 K, and heating to *T*_annealing_ = 343 K. The obtained crystals were subsequently analyzed as a function of temperature in the range: 273–373 K (M-*ortho*-F polymorph **1b**), 353–403 K (M-*ortho*-F polymorph **2b**), 243–373 K (M-*meta*-F polymorph **1a**), and 203–373 K (M-*meta*-F polymorph **2a**).

### Powder X-ray Diffraction

PXRD experiments were performed using a Rigaku-Denki D/MAX/RAPID II-R diffractometer equipped with a rotating silver anode and a two-dimensional image plate detector. The wavelength of the incident beam was 0.5608 Å. The samples were measured in glass capillaries. The two-dimensional diffraction pattern was corrected for background (subtracted empty capillary) and converted to a one-dimensional function of the diffraction intensity vs the scattering angle 2θ. The angular range covered by the experiment was 2–164°, but the range presented in the article was a range of 2–11° characterized by the occurrence of Bragg peaks.

### Single-Crystal X-ray Diffraction

The single-crystal X-ray experiments were performed at 100 K. The data were collected using a SuperNova four-circle kappa diffractometer with an Atlas CCD detector (formerly Agilent Technologies, currently Rigaku Oxford Diffraction). For the integration of the collected data, the CrysAlis^Pro^ software was used.^25^ The structures were solved using direct methods with the SHELXS-2013 software and the solutions were refined using the SHELXL-2019/2 program.^26^ Crystal, data collection, and refinement details for the crystal structures are gathered in Table S2.

## Results and Discussion

First, we characterize the thermal properties of M-*meta*-F and M-*ortho*-F using DSC. Both isomers were found to be in the glassy state, as confirmed by the presence of a glass transition, visible as a step change in heat capacity on collected thermograms (Figure 1a). The glass transition temperatures, *T*_g_, determined from the midpoint of heat capacity increment, are the same, i.e., *T*_g_ = 313 ± 1 K for M-*meta*-F and M-*ortho*-F. The DSC measurements were performed with a heating rate of 10 K/min; no crystallization was observed under these conditions. Subsequently, the molecular dynamics of both isomers were investigated using the BDS method.

Figure 1b,c shows the representative dielectric loss spectra ε″(*f*), collected at different temperatures for samples above and below *T*_g_. In the glassy state (Figure 1b), molecular mobility is determined by secondary relaxations. In general terms, secondary relaxations may reflect the intramolecular dynamics of molecular fragments or the dynamics of an entire molecule. The latter called Johari–Goldstein (JG) relaxations are frequently discussed as precursors of the cooperative dynamics associated with the glass transition.^27,28^ In the case of molecular rotors, intramolecular relaxations are of greatest importance, as the focal point of research is a molecular fragment called the rotor, performing rapid rotations in the environment created by the rest of the molecule. In the studied molecules, internal rotation was accomplished with a fluorophenyl unit. Due to the presence of a fluorine atom, rotor mobility leads to dipole moment changes. Thus, in a glassy state, the rotation of the fluorophenyl unit contributes to the dielectric response as a secondary relaxation mode (non-JG), which we assigned as the β_glass_ process.^29,30^ As shown in Figure 1b when the glassy sample is heated, the maximum of the β_glass_ process shifts toward higher frequencies. As the loss peak frequency is inversely related to the relaxation time, such behavior proves that the internal rotation of fluorophenylene units accelerates on heating. When the temperature reaches *T* > *T*_g_, a higher amplitude ε″(*f*) peak, α-process, appears on the spectrum (see Figure 1c). It corresponds to the structural relaxation process originating from the cooperative rearrangements of whole molecules and constitutes an intrinsic feature of supercooled liquids dynamics. One can see in Figure 1c that for the M-*ortho*-F at *T* = 353 K, a significant reduction in the ε″(*f*) peak amplitude occurs. It denotes the ongoing crystallization. The drop in ε″(*f*) peak amplitude is caused by the partial transformation of supercooled liquid into a crystalline phase where the whole molecule motions responsible for α-process are limited.

**Figure 2:**
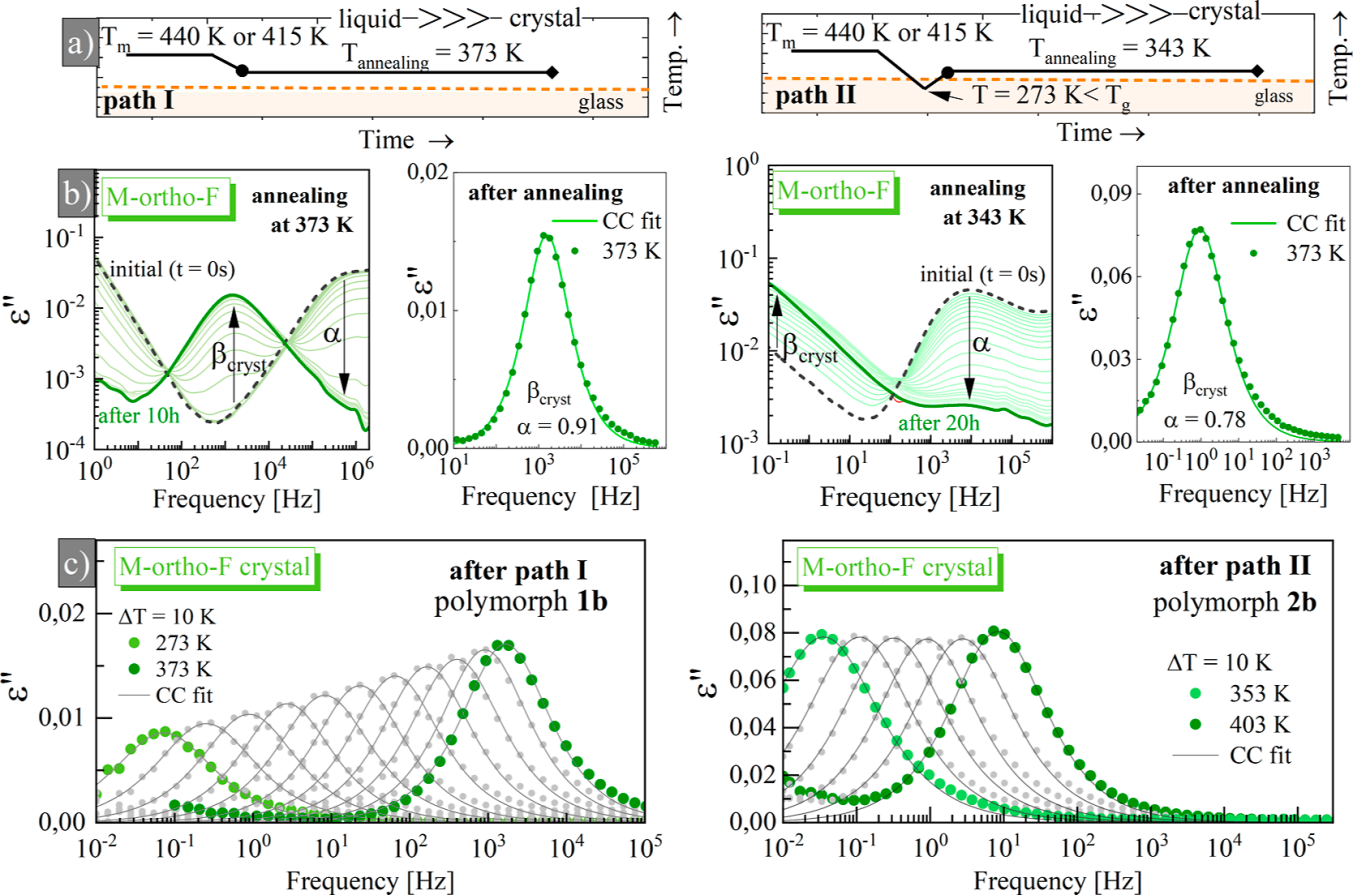
Look horizontally to see the BDS results for representative crystals of M-*ortho*-F after path I (first column) and after path II (second column). (a) Schematic representation of applied recrystallization protocol. (b) (Left panel) the progress of crystallization detected during isothermal annealing for selected polymorph. (Right panel) the effect of Cole–Cole (CC) fitting analysis with the value of corresponding α parameter. (c) Dielectric loss spectra collected for recrystallized samples showing the β_cryst_ processes at different temperatures. Similar data for M-*meta*-F can be found in the Supporting Information.

The observation of the recrystallization phenomena prompted us to perform time-dependent isothermal dielectric experiments to monitor the crystallization of the investigated isomers in situ. We applied two crystallization protocols intending to produce different polymorphs by cooling the melt to various temperatures. We expected that nucleation at different temperatures (i.e., near *T*_m_ and near *T*_g_) would yield different polymorphic forms.^31−35^ The overview of the two experimental protocols used in our study is shown in Figure 2a. The applied procedures include the following steps (i) melting the substance at *T* = *T*_m_, cooling to *T*_anealing_ > *T*_g,_ (path I) and (ii) melting the substance at *T* = *T*_m_, cooling to *T* < *T*_g_, and subsequent heating to *T*_anealing_ > *T*_g_ (path II). The main difference is the deeper cooling step and additional heating step in path II. The representative dielectric loss spectra ε″(*f*) collected during sample annealing for M-*ortho*-F are presented in Figure 2b. Data for M-*meta*-F are shown in Figure S7 in the Supporting Information. The corresponding ε′(*f*) data are shown in Figure S8.

As can be seen in Figure 2b, when the crystallization progresses, the α-process gradually disappears. Interestingly, when the crystallization was completed, the collected ε″(*f*) spectra were not featureless, which would be expected if all mobile dipoles were frozen in the crystalline lattice. Instead, simultaneously with the disappearance of the α-peak, a new relaxation process appears in the dielectric response; see Figure 2b,c, assigned as β_crys_, which indicates that the internal rotation of the fluorophenylene unit is not suppressed in a crystalline state. The same effect was reported before for similar crystals with an acetylene linker.^24^ Surprisingly, the pattern of the dielectric response depends on the applied crystallization protocol. For instance, for M-*meta*-F, the bimodal dielectric response was observed after path II, suggesting that a mixture of different polymorphs was formed (see a second column in Figure S7).

**Figure 3:**
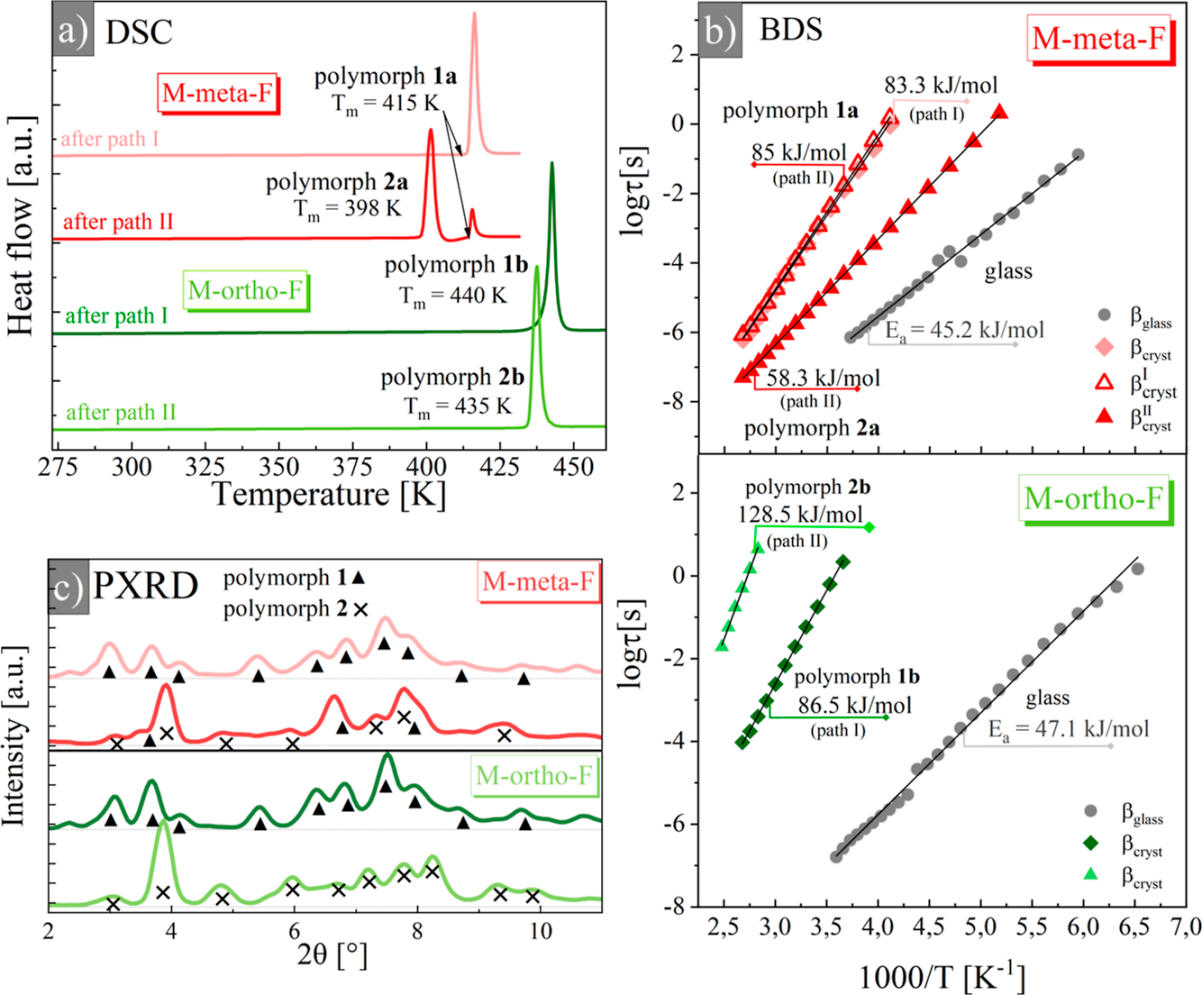
(a) Thermograms obtained on heating of recrystallized samples (10 K/min) obtained using different crystallization protocols (path I and path II) for M-*meta*-F (red lines) and M-*ortho*-F (green lines) isomers. (b) Relaxation map for different polymorphs and glass of M-*meta*-F and M-*ortho*-F. (c) Powder diffraction patterns of samples obtained using crystallization protocols like in diagram (a), symbols mark diffraction peaks which refer to polymorph **1** or **2**.

To confirm that the applied recrystallization protocols led to various polymorphic forms, we performed DSC measurements to characterize the melting points of the obtained crystals. Figure 3a presents thermograms obtained on heating of samples recrystallized on a BDS using different protocols. All the curves show an endothermic process associated with crystal melting. For the M-*meta*-F, we identified two polymorphs with *T*_m_ = 415 ± 1 K (assigned as polymorph **1a**) and *T*_m_ = 398 ± 1 K (polymorph **2a**). The recrystallization procedure according to path I resulted in polymorph **1a**, while path II led to the appearance of a lower melting point polymorph **2a**, which after melting promptly recrystallized to polymorph **1a** when heated at 10 K/min. To demonstrate that polymorph **1a** is not formed postmelting of polymorph **2a** but was formed during thermal annealing in BDS, the sample was heated with a faster heating rate of 30 K/min (see Figure S10). Then, the recrystallization to polymorph **1a** can be avoided and two endothermic processes corresponding to the melting of polymorphs **1a** and **2a** can be distinguished. For the M-*ortho*-F, we obtained polymorph **1b** with *T*_m_ = 440 ± 1 K following path I, and a lower melting point polymorph **2b** with *T*_m_ = 435 ± 1 K after path II. The structural evidence for the presence of various polymorphs in samples recrystallized using different protocols is given in Figure 3c where PXRD patterns for each recrystallized sample are compared. The diffractograms of M-*meta*-F and M-*ortho*-F show sharp Bragg peaks characterizing the crystalline structures of the investigated samples. Patterns of samples obtained after path I and path II are characterized by clear differences, indicating that we are dealing with different crystalline forms. A closer analysis of the M-*meta*-F crystal obtained via path II confirms that the obtained sample is a mixture of two polymorphs **1a** and **2a**, with the majority of the second. That observation helps to explain the two melting points and bimodal dielectric response.

To probe the rotational dynamics for each crystal, we measured the dielectric loss spectra as a function of the temperature when the recrystallization process was completed. The collected ε″(*f*) data are shown in Figures 2c and S7b. One can notice that ε″(*f*) spectra collected for crystals are much narrower than those presented in Figure 1b,c. To determine the characteristic relaxation times for internal rotation of the fluorophenyl unit in crystals (β_cryst_ process), we fitted the ε″(*f*) spectra depicted in Figures 2c and S7 using a CC function
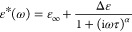
where ε_∞_ is the high-frequency permittivity, Δε is the dielectric strength, ω is the angular frequency, τ is the characteristic dielectric relaxation time, and α is a shape parameter which varies from 0 to 1 (for the Debye response with a single relaxation time).^36^ For the M-*meta*-F crystal with the bimodal response, we applied two CC functions (see Figure S7a, second column). For all crystalline samples, the α parameter is close to unity at high T and decreases on cooling, reflecting a departure from the ideal Debye model in terms of peak broadening (see Figure S9). Referring to the nomenclature typical for glass-forming liquids, it is denoted that rotational heterogeneity increases as the temperature decreases. In structurally ordered crystals, such effects were linked with the diversity of dipole–dipole interactions in the case of polar rotors,^37^ or temperature-dependent crystalline framework motion as was suggested for rotors in the metal–organic frameworks.^38^

The main outcome of CC analysis is presented in Figure 3b where the temperature dependence of characteristic relaxation times is shown. For all crystals, the relaxation times follow the linear Arrhenius relationship, allowing us to compare their rotational dynamic in terms of activation energy^39^
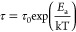
where τ_0_ is the pre-exponential factor linked to attempt frequency, *E*_a_ is the activation energy, *k* is Boltzmann’s constant, and *T* is the temperature (see Table S1 for fitting parameters). In all cases, the rotation barrier determined for the internal rotation of the fluorophenylene unit in the crystalline state was higher than that in the glassy state. This can be expected due to the lower density and larger free volume of glass compared to its crystalline counterpart. However, such regularity cannot be treated as a strict rule, as we recently showed for a rotor for which the barrier height in the crystal was halved.^24^ Here, for the β_glass_ process, the *E*_a_ values were *E*_a_ = 45.20 ± 0.72 and *E*_a_ = 47.1 ± 0.58 kJ/mol for M-*meta*-F and M-*ortho*-F, respectively. For the β_crys_ process, the barrier height for the M-*meta*-F sample recrystallized using path I was equal to 83.32 ± 0.65 kJ/mol (polymorph **1a**). For the sample recrystallized according to path II, which is a mixture of two polymorphs, we obtained two *E*_a_ values. For a slower component with a smaller amplitude *E*_a_ = 85.00 ± 0.42 kJ/mol, while for a faster higher-amplitude component *E*_a_ = 58.26 ± 0.08 kJ/mol. One can note in Figure 3b that relaxation times obtained for polymorph **1a** and those corresponding to a slower component after path II overlap perfectly. Thus, we assigned a relaxation with a smaller amplitude to polymorph **1a** and a faster higher-amplitude component to polymorph **2a**. In the M-*ortho*-F process with *E*_a_ = 86.47 ± 0.08 kJ/mol after path I corresponds to polymorph **1b** while this with *E*_a_ = 128.49 ± 2.66 kJ/mol observed for the crystal after path II is assigned to polymorph **2b**. These results demonstrated that rotors in crystals obtained using different crystallization protocols revealed various solid-state rotational performances. Depending on the polymorph, the barrier height for the internal rotation of the fluorophenylene unit varied substantially, showing that polymorph variation is an effective approach to tailor the rotor properties in the amphidynamic crystals.

**Figure 4:**
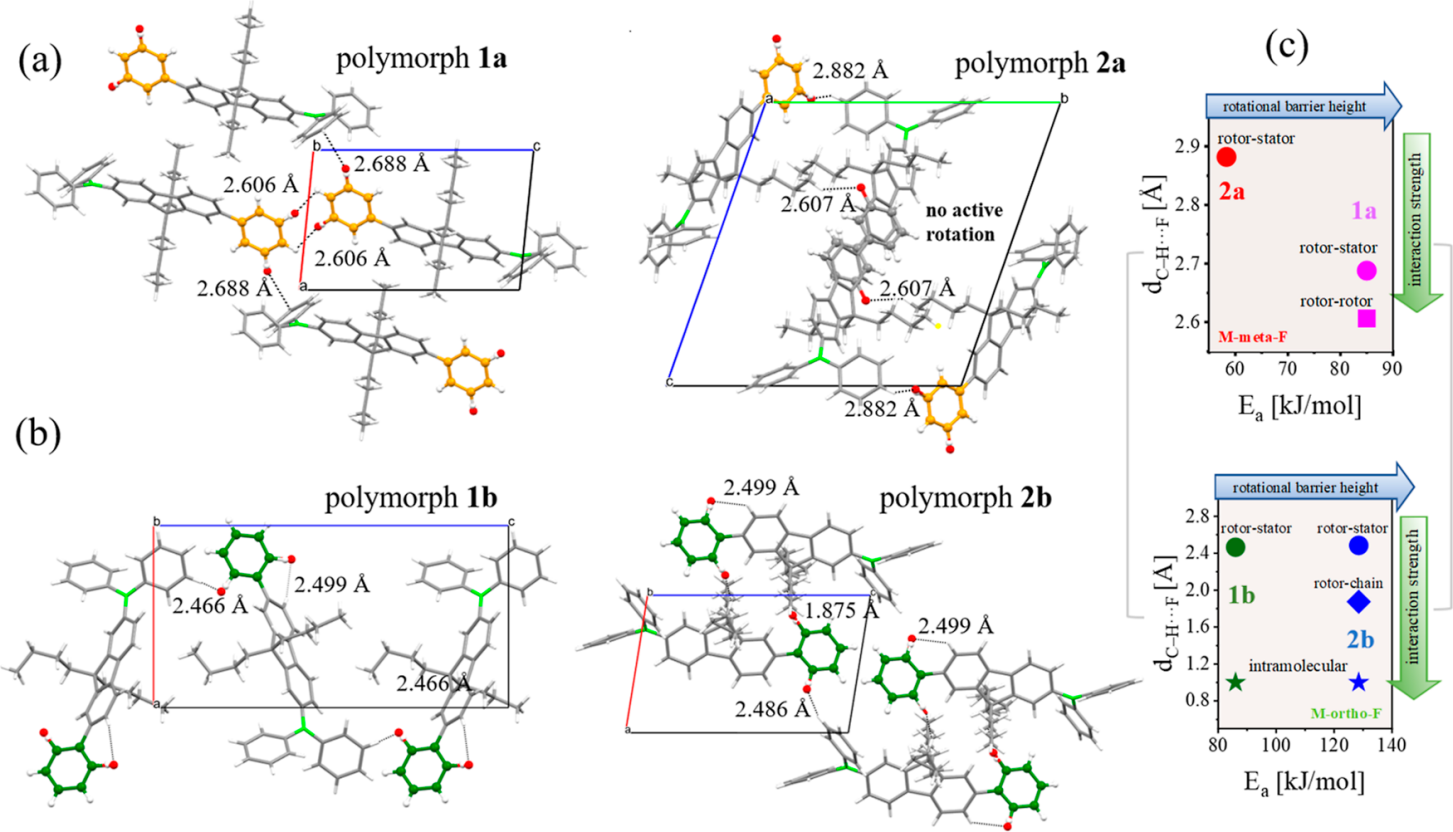
Packing geometry around the rotor with interatomic distances (in Å) for most relevant C–H···F contacts for M-*meta*-F (a) and M-*ortho*-F (b) polymorphs. The fluorophenylene units rotating in the crystalline interior are marked in orange (M-*meta*-F) or green (M-*ortho*-F). The unit cell axes are depicted. (c) Summary of results showing the relationship between the height of the rotational barrier, the number of possible interactions, and the distance between the donor and acceptor in the C–H···F contacts.

In our experiments, by maintaining the atomic composition in all tested samples and only modifying the packing arrangement, we created optimal conditions to identify the molecular factors determining the barrier height in each case. Single-crystal X-ray diffraction measurements provide precise structural information about each polymorph, which we could compare with BDS results. The crystallization protocols appear to be successful and allow us to grow single crystals of each polymorph suitable for structural analysis. In each sample, for most of the fluorophenyl units rotating in the crystalline interior, we observed that positional disorder and crystal refinement could be done only by assigning a fluorine location at two different sites, as shown in Figure 4 (F atoms are labeled in red) where packing geometry around the rotor and most relevant rotor-stator contacts are depicted (for thermal ellipsoid representation of structures see Figures S11–S14). For M-*meta*-F, polymorph **1a** and polymorph **2a** are packed in the same triclinic system with space group P-1 but the unit cell of polymorph **2a** has twice the volume of polymorph **1a** (see Table S2) and accommodates two molecules in the unit cell. The molecular arrangement and nature of intermolecular contacts vary among polymorphs. In polymorph **1a**, the M-*meta*-F molecules have an arrangement allowing both rotor–stator (*d*_C–H···F_ = 2.688 Å) and rotor–rotor (*d*_C–H···F_ = 2.606 Å) interactions, see Figure 4a. Regardless of the position of the rotating fluorine, the geometry of rotor surroundings allows the creation of C–H···F contacts with neighboring molecules, hindering the rotor’s mobility. In polymorph **2a**, the situation is more complex. In the unit cell, we have two molecules in which the rotor is located in different packing surroundings. The first population of rotor molecules can interact with the alkyl chains (*d*_C–H···F_ = 2.607 Å), and in this case, no positional disorder for the fluorine location was found. It denotes that rotor mobility remains suppressed in a crystalline lattice. The second population of rotor molecules interacts with one of the phenylamine rings, but the strength of interactions is weaker than in polymorph **1a** due to the larger distance between interacting atoms (*d*_C–H···F_ = 2.882 Å). During rotation, such contacts can be avoided at some fluorine locations as structures related to 180° rotation are not equivalent in terms of their ability to create rotor–stator contacts. Consequently, the internal rotation in polymorph **2a** occurs with a lower barrier height.

For M-*ortho*-F, following path I, polymorph **1b** was created with an orthorhombic unit cell and space group *P*2_1_2_1_2_1_; path II resulted in polymorph **2b** with a triclinic structure. Polymorph **1b** has a unit cell volume two times larger than polymorph **2b** (see Table S2 for details). Smaller rotational barriers had rotors moving in crystals with a larger volume of a unit cell; the same is true for M-*meta*-F polymorphs. The higher rotational barriers observed for M-*ortho*-F polymorphs in comparison to M-*meta*-F are due to the proximity of a fluorine atom to the stator’s fluorene unit, which allows the formation of intramolecular rotor–stator contacts, restricting the rotor’s mobility. The crystalline packing and pattern of relevant rotor–stator contacts for both polymorphs of M-*ortho*-F are shown in Figure 4b. For polymorph **1b**, in addition to intramolecular C–H···F interactions relevant for both M-*ortho*-F polymorphs (*d*_intra_ = 2.499 Å), intermolecular C–H···F contacts with one of the diphenylamine rings (*d*_C–H···F_ = 2.466 Å) affect rotor mobility. For polymorph **2b**, with the highest barrier detected, the molecular packing is different. During rotation, the fluorine atom can interact with one of the diphenylamine rings (*d*_C–H···F_ = 2.486 Å) but at the same time, the proximity of alkyl chains may hinder rotation, see the right panel in Figure 4b. Refined structures revealed some positional disorder for alkyl chains for polymorph **2b** in M-*ortho*-F. Since the terminal group of one of the alkyl chains can change its position, under certain conditions, a strong rotor-chain contact can emerge (with *d*_C–H···F_ = 1.875 Å), hindering rotation. Consequently, the largest barrier height discovered for this polymorph reflects the highest number of C–H···F contacts, allowing for developing rotor–stator interactions.

Our efforts to find a molecular justification for barrier height for the investigated fluorophenyl rotor have shown that the main factors are the interactions of fluorine with its immediate environment and differences in the pattern of C–H···F contacts determined by changes in the arrangement of atoms in individual crystal forms. It is apparent that the highest activation energy found for polymorph **2b** in M-*ortho*-F (*E*_a_ = 128.49 ± 2.66 kJ/mol) is related to the highest number of C–H···F interactions. The fewer rotor–stator contacts are created and the lower barriers are observed, as in the case of polymorph **2a** in M-*meta*-F with the lowest activation energy found (*E*_a_ = 58.26 ± 0.08 kJ/mol) and only one site favoring rotor–stator contacts. As shown in Figure 4c, the rotational barrier height correlates with the donor–acceptor distances of the involved C–H···F contacts. The proximity of the sizable core in the case of the M-*ortho*-F substitution, allowing the intramolecular C–H···F contacts, increased the barrier height compared to the *meta* isomer. The size of the unit cell is also important. For both isomers, the polymorph with lower activation energy had a larger unit cell. These results confirm the possibility of adjusting the internal dynamics of rotors via polymorph variation and show that the dynamics of a rotor in the crystalline interior can be effectively adjusted by finding a polymorph with optimal molecular arrangement limiting the possibility of rotor–stator interactions.

## Conclusions

In summary, we designed two molecular rotors with fluorophenylene rotors embedded in sizable crystals using an approach reported previously. By playing with the temperature during the crystallization procedure, we obtained various forms of an amphidynamic crystal, where the rotor moves in distinct packing environments with different patterns of rotor–stator interactions. Finally, we demonstrated that polymorph variation allows for the improvement of rotor properties and a reduction in barrier height by 30%. Our findings demonstrated that exploring the polymorphs of newly tested compounds presents a promising approach to identifying optimal rotational performance. Such polymorph screening can become an integral part of the search for new amphidynamic crystals, as it represents an opportunity to significantly improve rotational properties without synthesizing a new chemical entity.

## Data Availability

CCDC 2353733–2353736 contain the Supporting Information crystallographic data for this paper. These data can be obtained free of charge from The Cambridge Crystallographic Data Centre via: www.ccdc.cam.ac.uk/data_request/cif.

## Supporting Information Available

Chemical characterization of M-*meta*-F and M-ortho-F, dielectric investigation of crystallization on BDS, ε′(*f*) data illustrating crystallization progress for all polymorphs, analysis of shape parameter α_CC_ as a function of temperature, thermogram of M-*meta*-F with heating rate 30 K/min, structures of all polymorphs in thermal ellipsoid representation, and fitting parameters and structural parameters for single crystals (PDF)
Crystallographic data for all investigated polymorphs (CIF)

## Supplementary Material

jp4c04964_si_001.pdf

jp4c04964_si_003.cif
