# Supplementary material for: Engineering of Rotational Dynamics via Polymorph Manipulation
Source: J Phys Chem A. 2024 Dec 5;128(50):10758–65. doi: 10.1021/acs.jpca.4c04964 (PMC11665591; doi:10.1021/acs.jpca.4c04964)
Supplement: Supplementary file 1 — jp4c04964_si_001.pdf [file jp4c04964_si_001.pdf]

Supporting Information

Engineering of Rotational Dynamics via Polymorph Manipulation

Alfred Błażytko[1], Marzena Rams-Baron[1]*, Maria Książek[1], Joachim Kusz[1], Marek Matussek[2], Joanna Grelska[1], Marian Paluch[1]

[1]*August Chelkowski Institute of Physics, University of Silesia in Katowice, 75 Pulku Piechoty 1, 41- 500 Chorzow, Poland*
[2]*Institute of Chemistry, University of Silesia in Katowice, Szkolna 9, 40-006 Katowice, Poland*
*corresponding author: marzena.rams-baron@us.edu.pl

1. Chemical characterization of compounds

M-meta-F

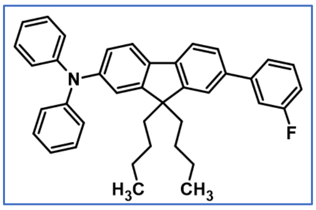

^{1}H NMR (500 MHz, Acetone-d$_6$) δ: 7.85 – 7.75 (m, 3H), 7.69 (dd, *J* = 7.9, 1.7 Hz, 1H), 7.62 – 7.58 (m, 1H), 7.55 – 7.48 (m, 2H), 7.35 – 7.29 (m, 4H), 7.23 – 7.20 (m, 1H), 7.15 – 7.10 (m, 5H), 7.08 – 7.02 (m, 3H), 2.16 – 1.93 (m, 4H), 1.17 – 1.04 (m, 4H), 0.74 – 0.66 (m, 10H). **^{13}C NMR** (126 MHz, CDCl$_3$) δ: 164.32, 162.37, 152.51, 151.52, 148.05, 147.47, 144.13, 144.07, 140.96, 138.01, 135.73, 130.29, 130.23, 129.28, 126.11, 124.01, 123.52, 122.79, 122.69, 121.31, 120.63, 119.54, 119.32, 114.09, 113.92, 113.89, 113.72, 55.19, 40.11, 26.17, 23.10, 13.99. **HRMS** (ESI): calcd. for $C_{39}H_{38}FN$ [M$^+$] 539.2988; found 539.2991.

M-ortho-F

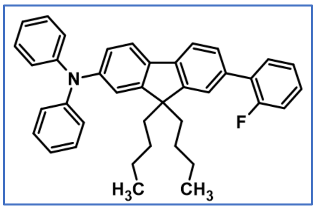

^{1}H NMR (500 MHz, Acetone-d$_6$) δ: 7.83 (d, *J* = 7.8 Hz, 1H), 7.77 (d, *J* = 8.1 Hz, 1H), 7.65 – 7.55 (m, 3H), 7.46 – 7.39 (m, 1H), 7.35 – 7.24 (m, 6H), 7.23 – 7.20 (m, 1H), 7.14 – 7.02 (m, 7H), 2.00 – 1.92 (m, 4H), 1.16 – 1.07 (m, 4H), 0.75 – 0.68 (m, 10H). **^{13}C NMR** (126 MHz, CDCl$_3$) δ: 160.91, 158.94, 152.54, 150.84, 148.06, 147.34, 140.56, 135.91, 133.73, 130.86, 130.84, 129.75, 129.65, 129.24, 128.71, 128.65, 127.86, 127.84, 124.40, 124.37, 123.95, 123.54, 123.52, 123.49, 122.61, 120.56, 119.36, 119.10, 116.31, 116.12, 55.12, 39.99, 26.14, 23.07, 13.95. **HRMS** (ESI): calcd. for $C_{39}H_{38}FN$ [M$^+$] 539.2988; found 539.2991.

Mass	RA	Calc. Mass	mDa	PPM	DBE	Formula	i-FIT	i-FIT Norm	Fit Conf %	C	H	N	F
539.2991	100.00	539.2988	0.3	0.6	21.0	C39 H38 N F	1001.1	n/a	n/a	39	38	1	1
540.3029	59.95	540.3067	-3.8	-7.0	20.5	C39 H39 N F	929.9	n/a	n/a	39	39	1	1

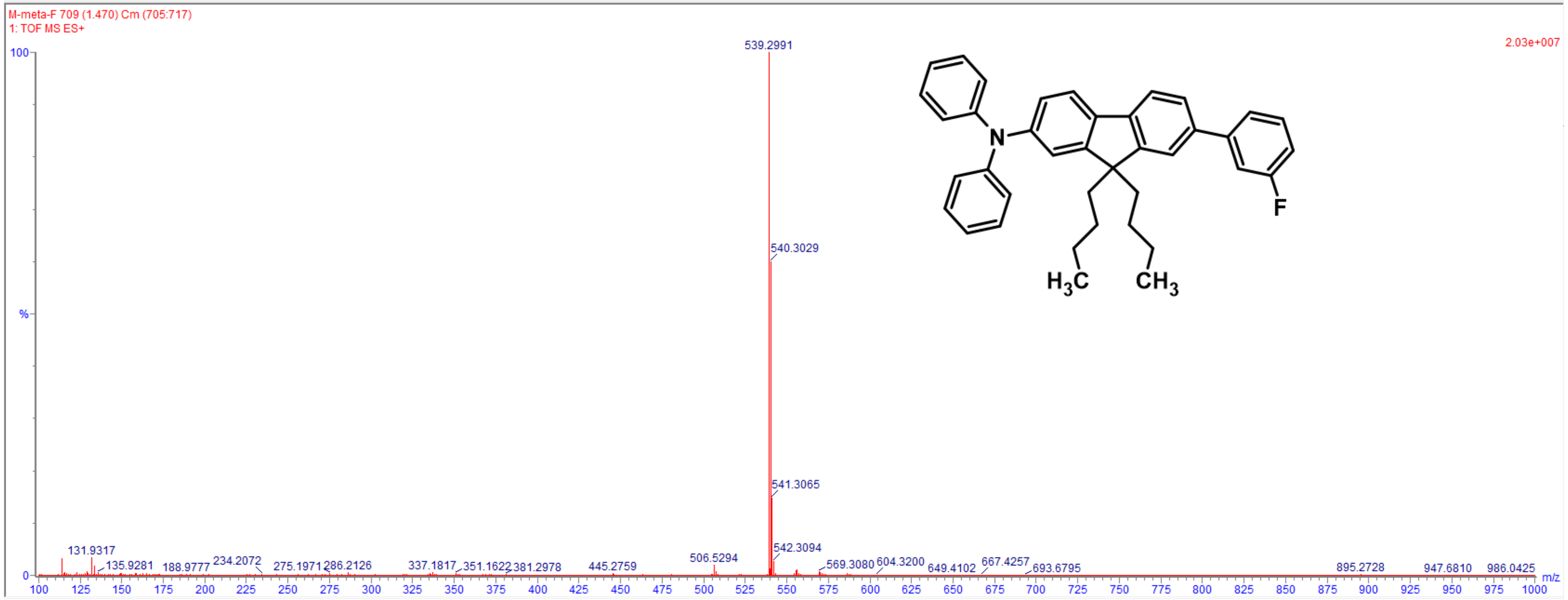

Figure S1: HRMS spectrum of M-meta-F

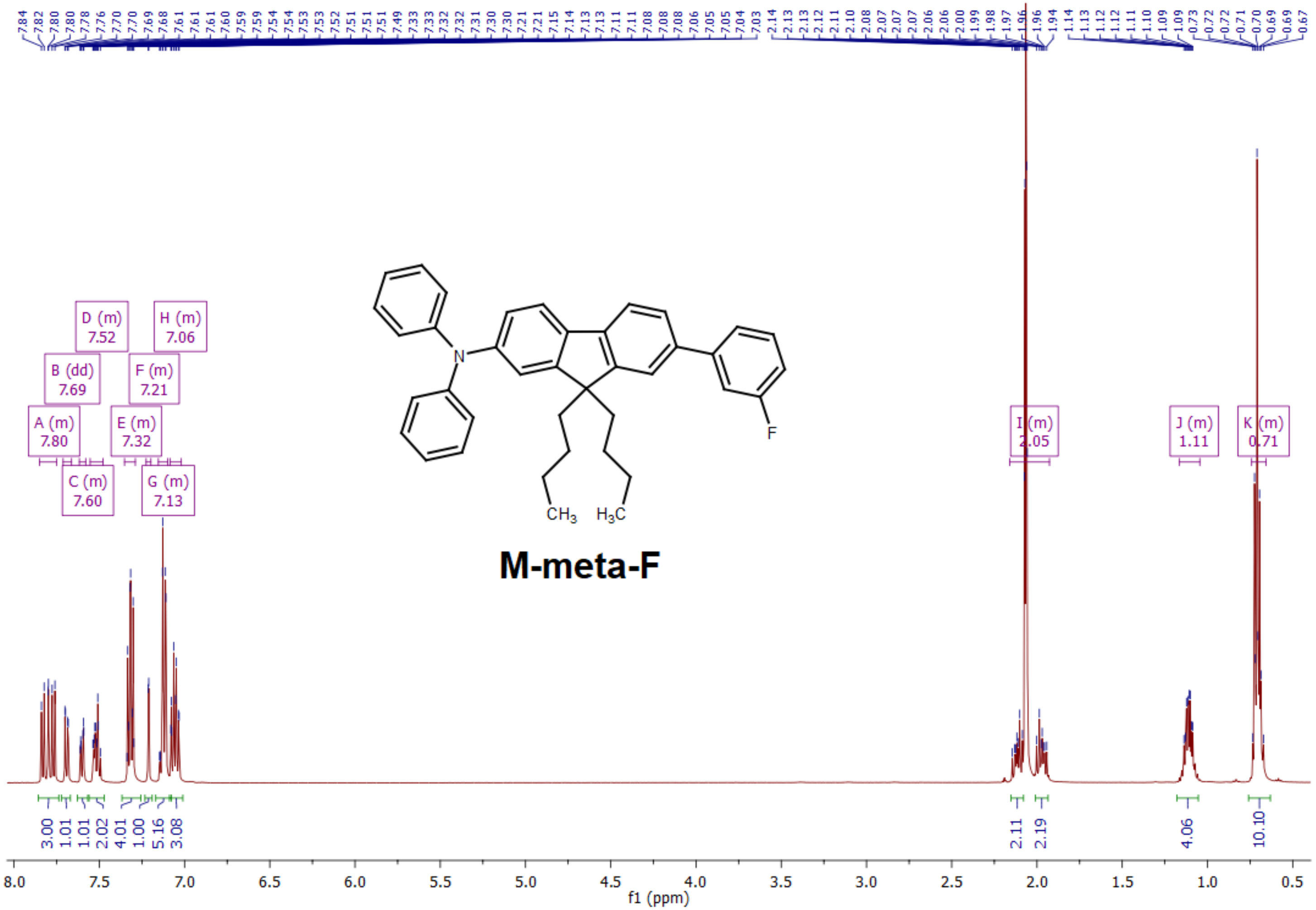

Figure S2: ^{1}H NMR spectrum of M-meta-F

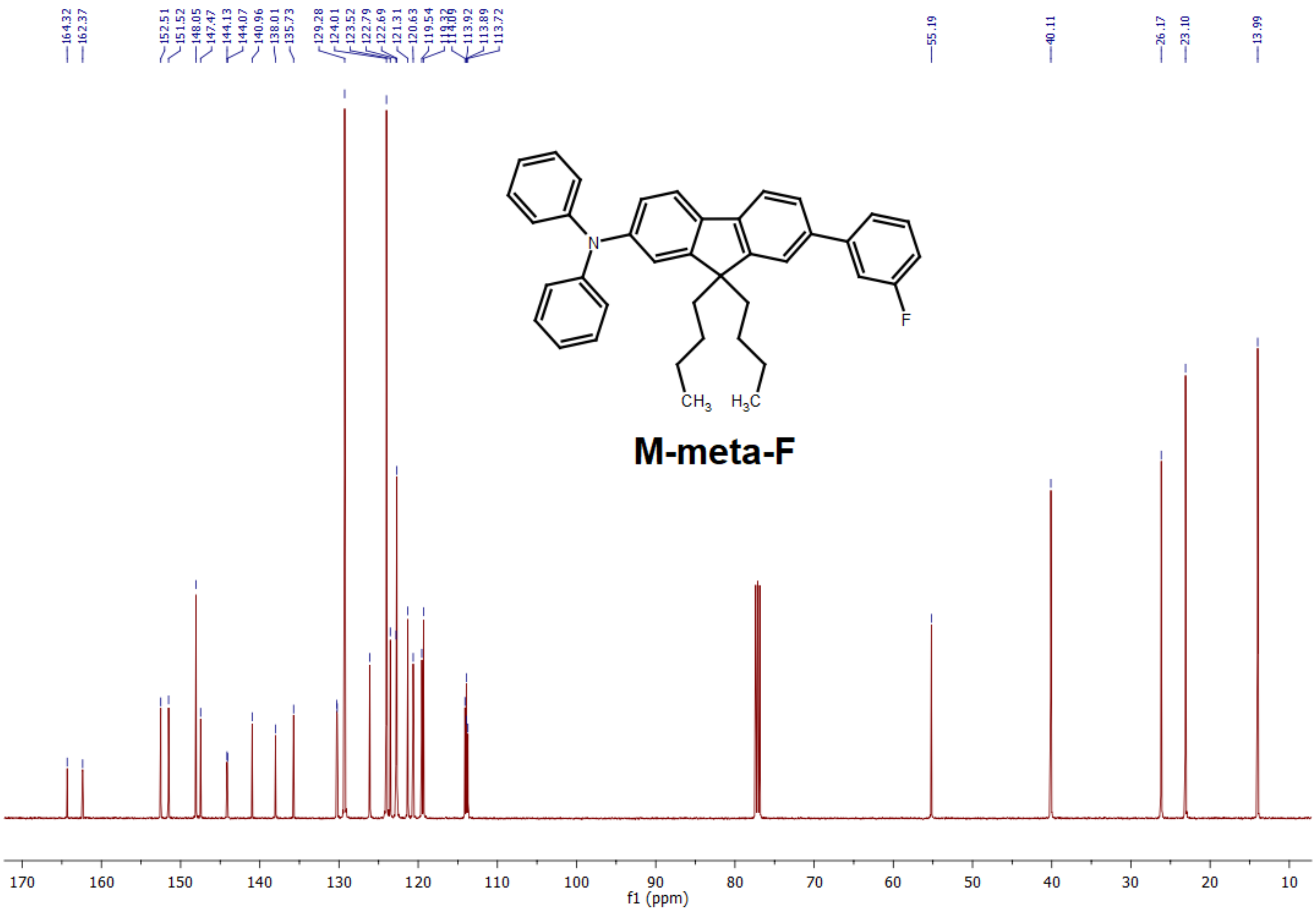

Figure S3: ^{13}C NMR spectrum of M-meta-F

Mass	RA	Calc. Mass	mDa	PPM	DBE	Formula	i-FIT	i-FIT Norm	Fit Conf %	C	H	N	F
539.2991	100.00	539.2988	0.3	0.6	21.0	C39 H38 N F	998.2	n/a	n/a	39	38	1	1
540.3031	55.25	540.3067	-3.6	-6.7	20.5	C39 H39 N F	949.4	n/a	n/a	39	39	1	1

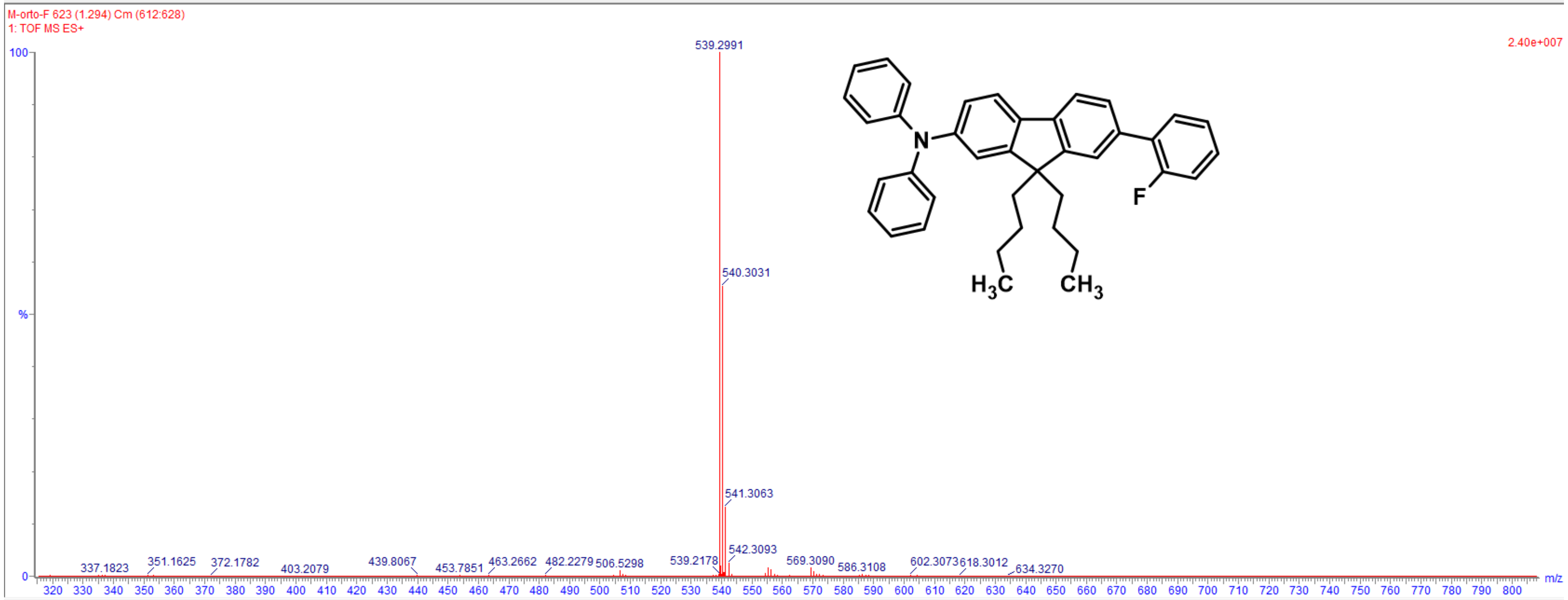

Figure S4: HRMS spectrum of M-ortho-F

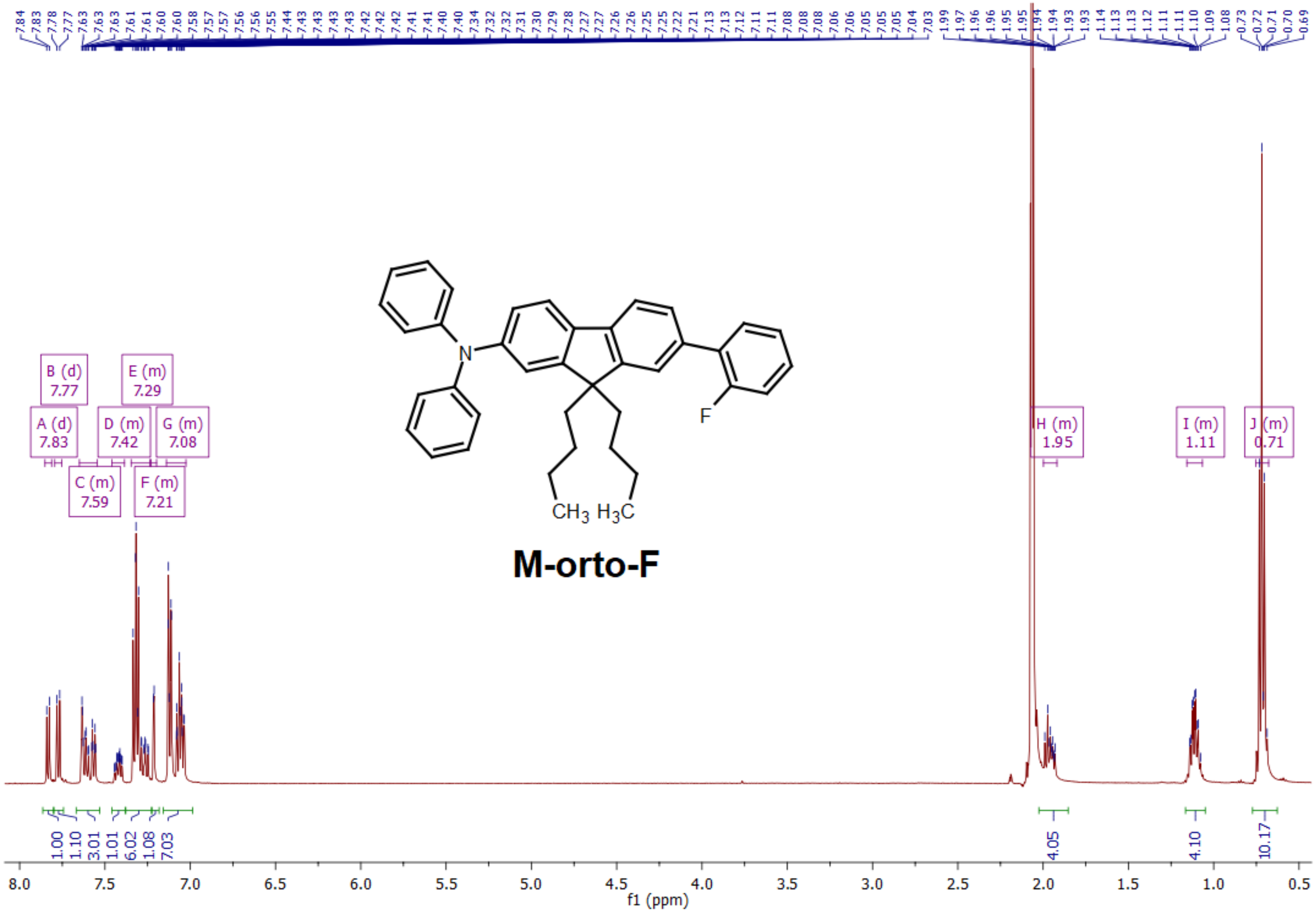

Figure S5: ^{1}H NMR spectrum of M-ortho-F

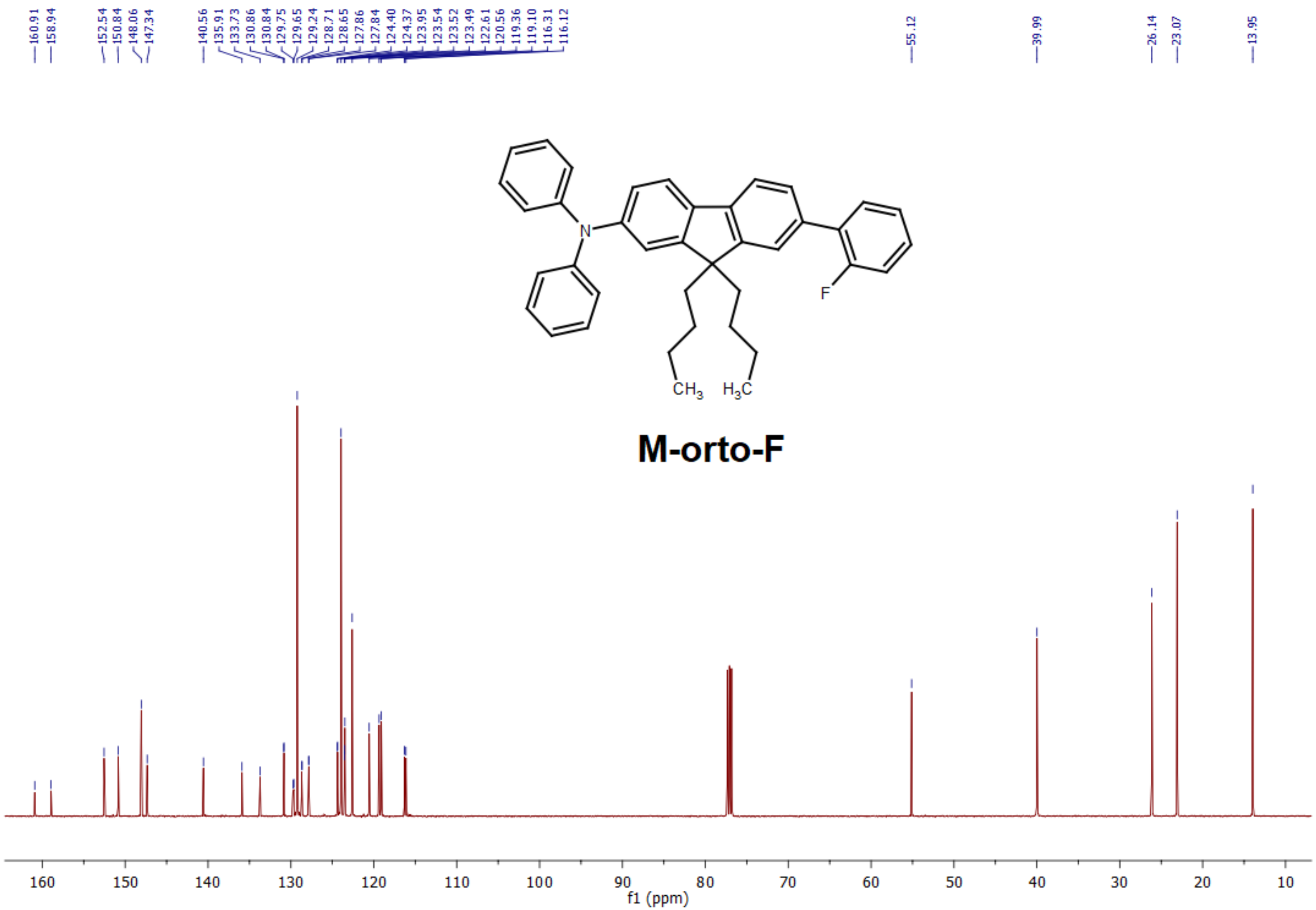

Figure S6: ^{13}C NMR spectrum of M-ortho-F

2. Supporting graphics and tables

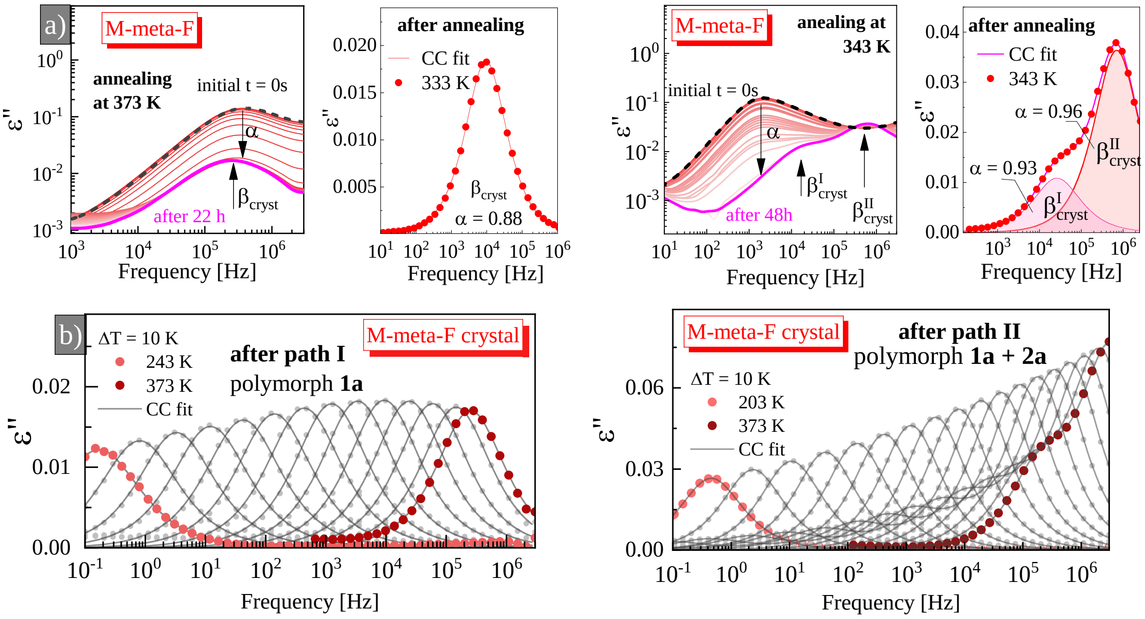

Figure S7: a) The progression of crystallization for not presented in main text M-meta-F polymorphs. Right column show selected spectrum with Cole-Cole function and shape parameters. b) Dielectric spectra showing the obtained β_{cryst} processes at different temperatures.

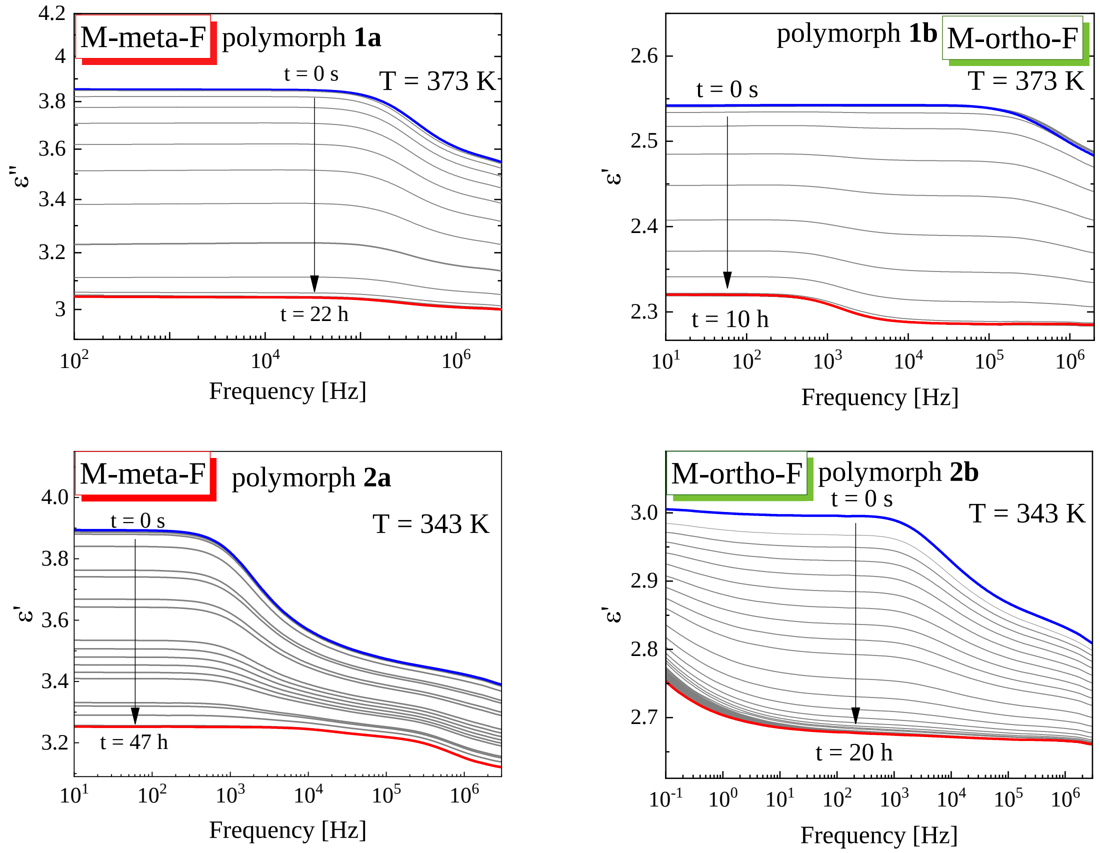

Figure S8: ε’ data for crystallization progress of all polymorphs.

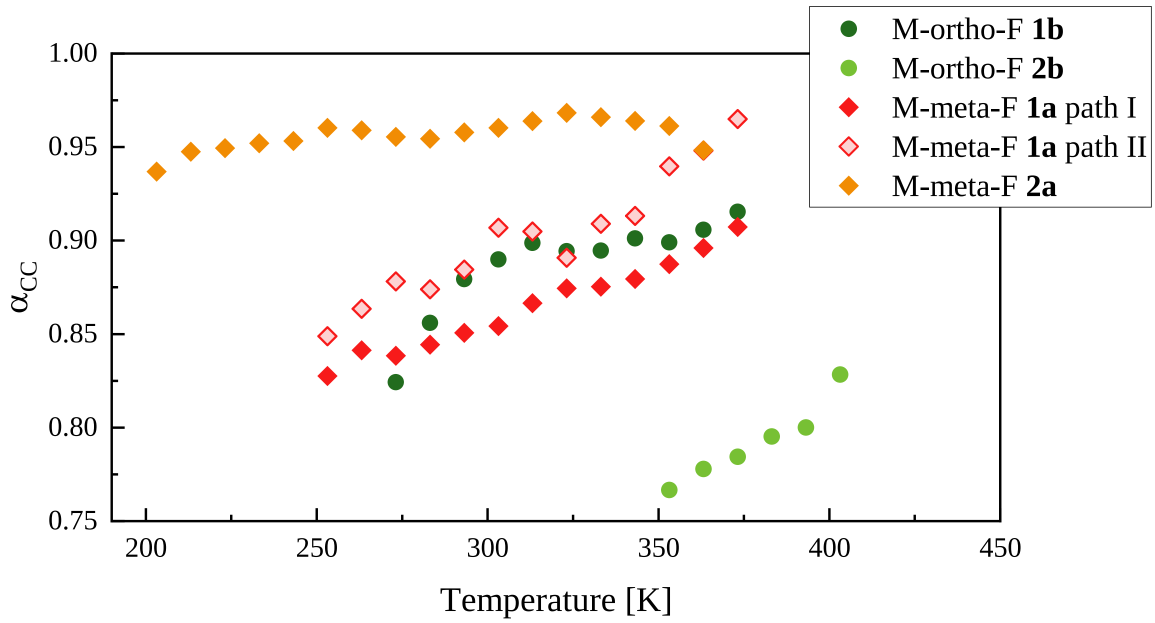

Figure S9: Changes in the shape parameter α of the CC function as a function of temperature.

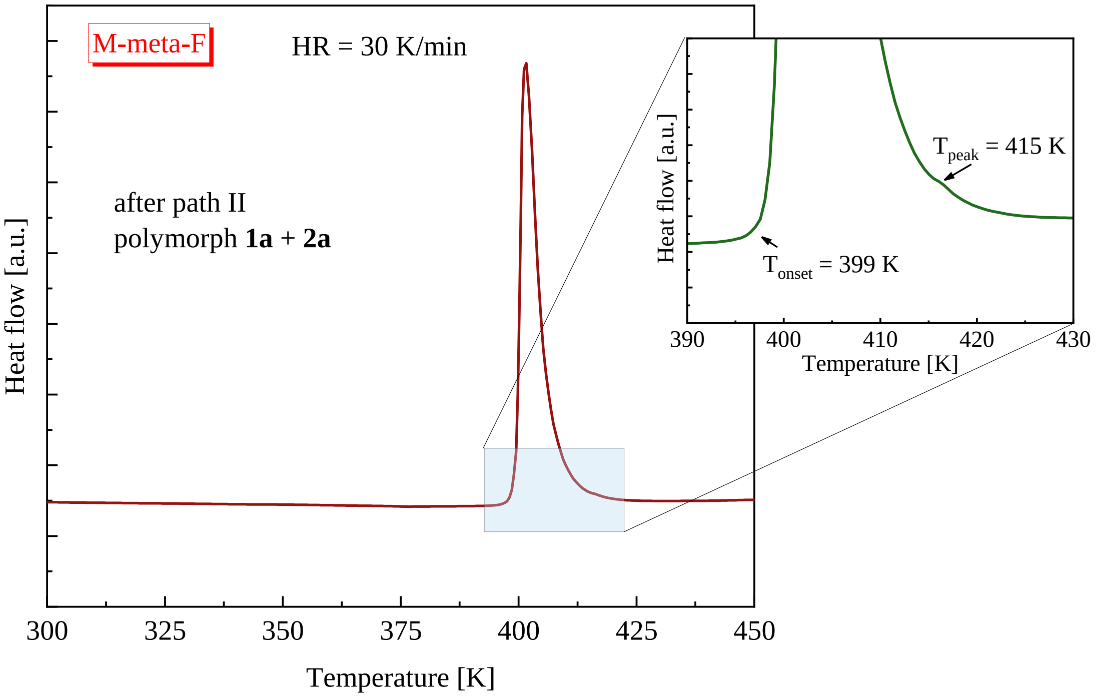

Figure S10: Thermogram obtained on heating of recrystallized M-meta-F after path II with 30 K/min heating rate.

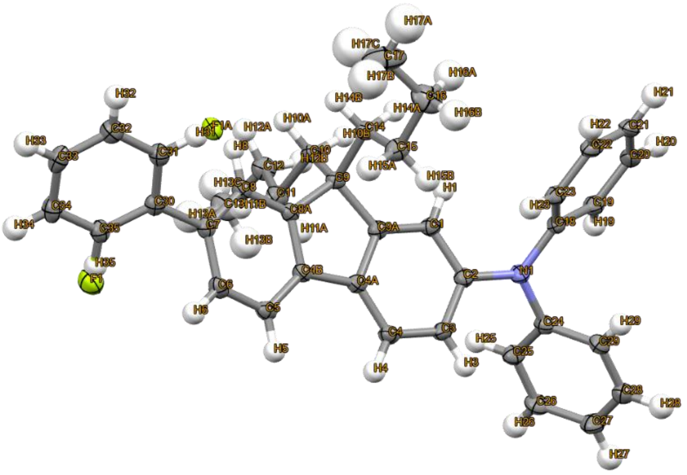

Figure S11: M-ortho-F polymorph **1b** structure in thermal ellipsoid representation.

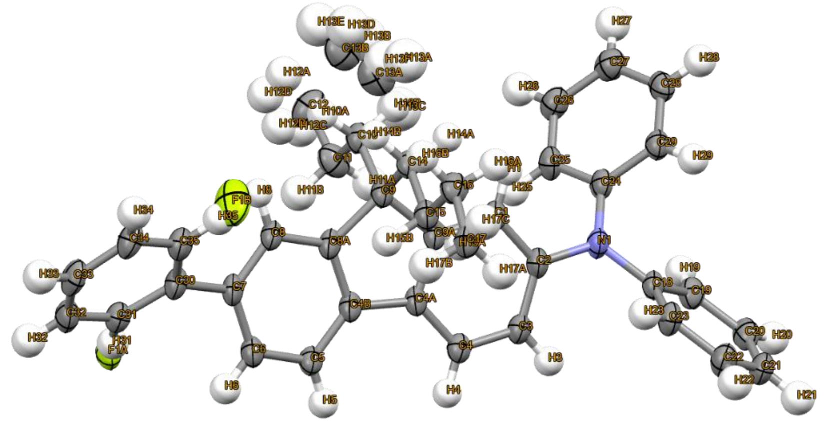

Figure S12: M-ortho-F polymorph **2b** structure in thermal ellipsoid representation.

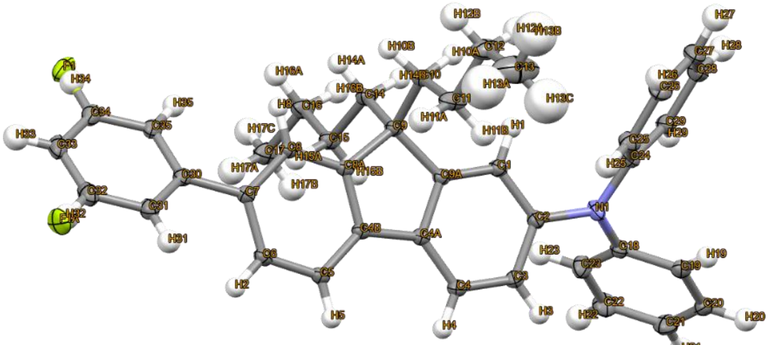

Figure S13: M-meta-F polymorph **1a** structure in thermal ellipsoid representation.

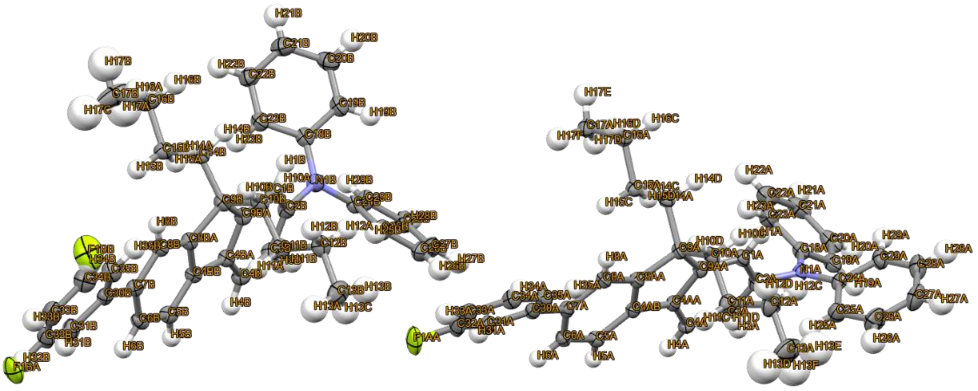

Figure S14: M-meta-F polymorph **2a** structure in thermal ellipsoid representation.

Table S1: Fit parameters of Arrhenius function for glasses and crystals.

		E_a [kJ/mol]	logτ [s]
**M-meta-F**	Glass	45.20 ± 0.72	-14.98 ± 0.18
	Polymorph **1a** path I	83.32 ± 0.65	-17.85 ± 0.11
	Polymorph **1a** path II	85.00 ± 0.42	-18.06 ± 0.07
	Polymorph **2a**	58.26 ± 0.08	-15.48 ± 0.01
**M-ortho-F**	Glass	47.10 ± 0.58	-15.61 ± 0.15
	Polymorph **1b**	86.47 ± 0.08	-16.16 ± 0.52
	Polymorph **2b**	128.49 ± 2.66	-18.32 ± 0.37

Table S2: Structural parameters of obtained single crystals of M-meta-F and M-ortho-F.

	**M-meta-F**		**M-ortho-F**	
	**polymorph 1a**	**polymorph 2a**	**polymorph 1b**	**polymorph 2b**
CCDC	2353733	2353734	2353736	2353735
T/K	100			
Chemical formula	$C_{39}H_{38}FN$			
Formula Mass	539.70			
Wavelength/Å	0.71073		1.54184	
Crystal system	Triclinic		Orthorhombic	Triclinic
Space group	P -1		P 2_1 2_1 2_1	P -1
Z	2	4	4	2
Unit cell dimensions				
a/Å	9.2598(2)	10.1561(3)	10.7172(2)	9.1595(9)
b/Å	12.6652(4)	17.1667(4)	13.3801(3)	12.6777(12)
c/Å	14.3895(4)	18.3720(4)	20.9751(5)	14.2485(9)
α/°	72.700(3)	107.171(2)	90	104.837(7)
β/°	89.726(2)	97.397(2)	90	93.252(7)
γ/°	71.324(2)	97.617(2)	90	109.944(9)
Unit cell volume/Å^3	1518.72(8)	2985.28(13)	3007.77(12)	1484.4(2)
F(000)	576	1152	1152	576
D_x /Mg m^{-3}	1.180	1.201	1.192	1.207
μ /mm^{-1}	0.072	0.073	0.559	0.566
Theta range for data collection/^{o}	2.958 to 36.357	2.951 to 36.534	3.919 to 73.720	3.250 to 73.673
Range of *h*, *k*, *l*	$-15 \leq h \leq 13$<br>$-20 \leq k \leq 20$<br>$-23 \leq l \leq 21$	$-16 \leq h \leq 16$<br>$-27 \leq k \leq 23$<br>$-21 \leq l \leq 30$	$-13 \leq h \leq 13$<br>$-16 \leq k \leq 14$<br>$-21 \leq l \leq 26$	$-11 \leq h \leq 10$<br>$-10 \leq k \leq 15$<br>$-17 \leq l \leq 17$
No. of measured reflections	23308	45564	19730	8491
No. of independent reflections	13469	26507	5905	5229
R_{int}	0.0292	0.0405	0.0330	0.0427
Data / restraints / parameters	13469 / 0 / 382	26507 / 0 / 754	5905 / 0 / 382	5229 / 0 / 393
Goodness-of-fit on F^2	1.035	1.022	1.047	1.054
Final R_1 values ($I > 2\sigma(I)$)	0.0551	0.0584	0.0343	0.0910
Final $wR(F^2)$ values ($I > 2\sigma(I)$)	0.1396	0.1370	0.0823	0.2562
Final R_1 values (all data)	0.0829	0.1016	0.0383	0.1031
Final $wR(F^2)$ values (all data)	0.1648	0.1729	0.0850	0.2822
Largest diff. peak and hole/eÅ^3	0.569 and -0.273	0.494 and -0.309	0.214 and -0.188	0.414 and -0.454
